# On the Restriction of Variolous Inoculation

**Published:** 1805-08-01

**Authors:** 


### On the Restriction of Variolous Inoculation.

*To the Editors of the Medical and Physical Journal.*

Gentlemen,

Among the various causes which have tended to bring discredit on the practice of vaccine inoculation, one is the little attention which has been paid to producing the genuine vaccine vesicle. It is not much to be wondered at that errors were committed at the commencement of this practice; but there was reason to hope, that in a course of seven years every medical practitioner would be sufficiently acquainted with the process to guard against being guilty of any very glaring blunder.

I am sorry to find that much misconduct and want of due care are still to be met with. A boy has lately been brought to London, secured, as his friends thought, from the danger of small-pox; for he had been inoculated with cow-pox matter in March last, by a surgeon in the country. A few days ago an eruption of papulæ appeared upon him, which his parents mistook for the small-pox. This led to an examination of his arms, on which there remain not the least vestige of inoculation; no mark, no indentation is to be seen, even through a magnifying glass. His parents were of course informed, that though the present eruption was not variolous, their son was by no means secure from the infection of that dreadful malady.

This morning's papers announce a Resolution of the Royal Jennerian Society to endeavour, either by application to parliament or by other means, to procure the restriction of the practice of variolous inoculation, or the adoption of such measures as may be found necessary to prevent the spreading of the casual small-pox from such inoculations. The utility of some measures for preventing the propagation of this disease has been often urged. Dr. Haygarth, some years ago, published a very valuable work on the possibility of exterminating the small-pox by proper regulations, and by preventing the diffusion of the contagion. It is evident that the introduction of the Jennerian inoculation would render such a measure much more practicable. Restrictions and regulations have been wisely enjoined by the legislature to prevent the introduction and communication of the plague and yellow fever, and and measures have been advantageously adopted on a more confined scale to prevent the spreading of infectious fevers, &c. in close and crowded neighbourhoods.

If the Committee of the Jennerian Society possesses, or can procure, any well authenticated instances of the diffusion of variolous contagion from those who have been inoculated, the publication of such proofs, specifying those who had the disease severely, and those who died of it, would tend very much to convince the magistrates, public officers, and the people at large, of the propriety and necessity of such restrictions. It would probably be no very difficult undertaking, particularly in the villages round London, to trace the small-pox through many patients to its origin in an inoculated child.

How dreadful a responsibility rests upon those who carelessly and wantonly expose their families and neighbours to the infection of this most loathsome and malignant enemy of the human race.

*July* 10, 1805. S. M.

